# When Marrow Mimics Malignancy: Tumefactive Hepatic Extramedullary Hematopoiesis Presenting as a Hypervascular Liver Mass

**DOI:** 10.7759/cureus.112274

**Published:** 2026-07-08

**Authors:** Luis Erick Cardona-Rodriguez, Mariana Andrea Ortiz-García, Ivan Omar Gómez-Figueroa, Sofia De-La-O-Villalobos, Raúl Isaí Castillo-Cabrera

**Affiliations:** 1 Gastroenterology, Centro Medico Nacional 20 de Noviembre, Mexico City, MEX; 2 Internal Medicine, Hospital Regional Presidente Lázaro Cárdenas, Instituto de Seguridad y Servicios Sociales de los Trabajadores del Estado (ISSSTE), Chihuahua, MEX; 3 Hematology, Centro Medico Nacional 20 de Noviembre, Mexico City, MEX

**Keywords:** ca 19-9 elevation, extramedullary hematopoiesis, hepatic pseudotumor, hepatocellular carcinoma, tumefactive extramedullary hematopoiesis

## Abstract

Extramedullary hematopoiesis (EMH) is the production of blood cells outside the bone marrow, usually occurring as a compensatory response to hematologic stress or marrow dysfunction. Although EMH most commonly presents as diffuse tissue infiltration, it can rarely manifest as a localized solid mass, known as tumefactive EMH, creating a significant diagnostic challenge by mimicking benign or malignant lesions on non-invasive imaging. We report the case of a 47-year-old male with cirrhosis secondary to autoimmune hepatitis, receiving mycophenolic acid, who presented with severe symptomatic anemia, profound jaundice, hepatosplenomegaly, and markedly elevated carbohydrate antigen 19-9 (CA 19-9) levels. Dynamic abdominal CT revealed a 63-mm hypervascular ovoid mass in hepatic segment VIII, showing heterogeneous arterial-phase enhancement and relatively decreased attenuation on the delayed/parenchymal phase, raising strong suspicion for hepatocellular carcinoma or intrahepatic cholangiocarcinoma. Given the suspicious radiological and biochemical findings, a percutaneous liver biopsy was performed. Histopathological evaluation demonstrated sinusoidal and perisinusoidal hematopoietic elements with megakaryocytic and erythroid differentiation. Immunohistochemistry demonstrated CD61-positive megakaryocytic elements and glycophorin A-positive erythroid precursors, supporting hepatic EMH and ruling out hepatocellular carcinoma, cholangiocarcinoma, or metastatic malignancy in the analyzed tissue. Subsequent bone marrow evaluation showed mild reticulin fibrosis and findings suggestive of a possible early-stage myeloproliferative neoplasm; however, definitive molecular and cytogenetic characterization could not be completed, and diagnostic criteria for a definitive hematologic disorder were not fulfilled. This case highlights that tumefactive hepatic EMH can radiologically and biochemically simulate hepatobiliary malignancy, particularly in patients with chronic liver disease, cholestasis, and elevated CA 19-9. It underscores the essential role of histopathological confirmation in preventing inappropriate oncological management and illustrates that focal hepatic EMH should prompt evaluation for a possible underlying hematologic disorder without assuming a definitive myeloproliferative neoplasm.

## Introduction

Extramedullary hematopoiesis (EMH) is defined as the production of blood cells outside of the bone marrow. More specifically, it is characterized by the presence of two or three hematopoietic lineages (myeloid, erythroid, and megakaryocytic cells) at sites other than bone marrow. It represents a compensatory mechanism triggered by hematopoietic disorders, insufficient hematopoietic compensation, or other pathological stress conditions such as infection, advanced tumors, anemia, and metabolic stress [1-2]. In this context, EMH can be classified as a passive mechanism that occurs when the primary bone marrow niche becomes inhospitable or fails to sustain normal hematopoiesis, prompting the emigration and homing of hematopoietic stem and progenitor cells to alternative peripheral organs [3]. EMH typically occurs in organs that were actively involved in fetal hematopoiesis, including the liver, spleen, and lymph nodes. Large-scale cohort data reinforce this distribution, demonstrating that even in non-myeloproliferative neoplasm settings, the spleen (53%) and the liver (25%) remain the two most frequently involved anatomical sites. Less commonly, it can occur in the mediastinum, paraspinal/paravertebral region, adrenal glands, lung, pleura, gastrointestinal tract, kidney, prostate, breast, and other sites [4-6].

In terms of macroscopic presentation, EMH typically manifests as diffuse tissue infiltration; however, it can occasionally develop as a localized, solid mass. This mass-forming variant, known as tumefactive EMH, creates a significant diagnostic challenge because it frequently resembles malignant neoplasms on non-invasive imaging [2, 7, 8]. Hepatic tumefactive EMH is particularly challenging because it may present as a focal liver lesion with imaging features that overlap with a broad spectrum of benign and malignant hepatic lesions, including focal nodular hyperplasia, hepatic adenoma, hepatocellular carcinoma, intrahepatic cholangiocarcinoma, and metastatic disease. In this setting, histopathological confirmation is often essential to establish a definitive diagnosis, exclude malignancy, and prevent inappropriate management [7,8].

## Case presentation

A 47-year-old male with a history of chronic liver disease secondary to autoimmune hepatitis, previously classified as cirrhosis, and receiving mycophenolic acid 1 g/day, presented with asthenia, adynamia, dizziness, palpitations, progressive dyspnea, and generalized jaundice. Upon admission, the patient was hemodynamically stable, with a blood pressure of 108/62 mmHg, a heart rate of 64 beats per minute, a respiratory rate of 18 breaths per minute, and an oxygen saturation of 98% on room air. Physical examination was remarkable for mucocutaneous jaundice, hepatomegaly, and splenomegaly.

Initial laboratory studies showed severe anemia, with a hemoglobin level of 5.7 g/dL, and thrombocytopenia of 77,000/µL. The anemia workup showed a haptoglobin level of 32 mg/dL, a corrected reticulocyte count of 0.9%, and a negative direct antiglobulin test (direct Coombs test). Liver function tests showed aspartate aminotransferase of 93 U/L, alanine aminotransferase of 41 U/L, total bilirubin of 24.7 mg/dL, direct bilirubin of 18.24 mg/dL, indirect bilirubin of 6.46 mg/dL, alkaline phosphatase of 194 U/L, and gamma-glutamyl transferase of 540 U/L. The calculated R-factor was 0.6, compatible with a predominantly cholestatic pattern. Tumor markers showed a serum alpha-fetoprotein level of 6 ng/mL and carbohydrate antigen 19-9 (CA 19-9) of 637 U/mL. As part of the initial supportive management for severe symptomatic anemia, the patient received two units of packed red blood cells.

As part of the initial workup for jaundice and hepatomegaly, an abdominal ultrasound was performed, which identified a focal hepatic lesion. Subsequently, a dynamic liver CT scan was performed, showing a 63-mm ovoid mass located in hepatic segment VIII, which was isodense on the unenhanced phase, with predominant peripheral heterogeneous enhancement during the late arterial phase and relatively decreased attenuation on the delayed/parenchymal phase (Figure 1). 

**Figure 1:**
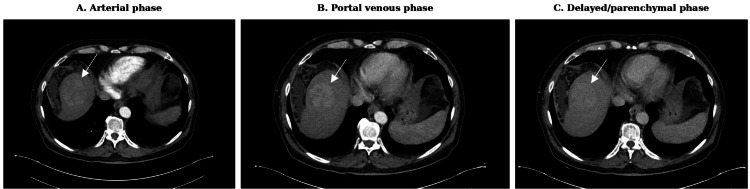
Dynamic contrast-enhanced abdominal CT Axial images show a 63-mm ovoid lesion in hepatic segment VIII. The lesion demonstrates heterogeneous enhancement on the arterial phase (A, white arrow), a persistent heterogeneous appearance on the portal venous phase (B, white arrow), and relatively decreased attenuation on the delayed/parenchymal phase (C, white arrow), radiologically mimicking a primary hepatic malignancy.

In the setting of chronic liver disease, marked cholestasis, elevated CA 19-9, normal alpha-fetoprotein, and a hepatic lesion with suspicious radiological features, it was deemed necessary to rule out hepatocellular carcinoma, intrahepatic cholangiocarcinoma, or another primary or metastatic hepatic neoplasm. For this reason, a percutaneous liver biopsy was performed to obtain a definitive histopathological diagnosis.

Histopathological evaluation of the hepatic lesion showed EMH associated with cholestasis, evidence of hepatocyte regeneration, and mild macrovesicular steatosis, estimated at approximately 5% (Figures 2, 3).

**Figure 2:**
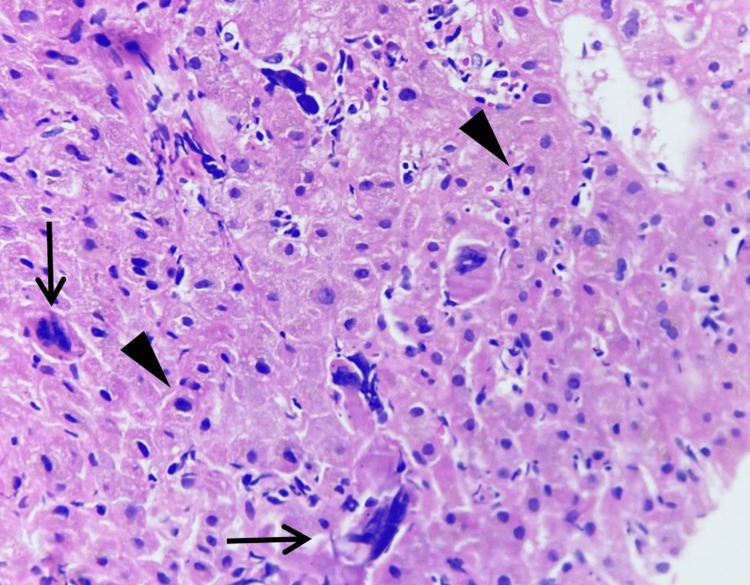
Histological section of the liver parenchyma (H&E, high-power view). The image shows a partially preserved hepatic architecture with diffuse sinusoidal expansion and hypercellularity due to immature hematopoietic elements. Small aggregates of immature erythroid precursors (basophilic normoblast-like cells) measuring 14-16 µm with dense chromatin are identified within the sinusoids (black arrowheads), alongside scattered large cells (40-50 µm) with hyperlobated nuclei, morphologically compatible with megakaryocytes (black arrows).

**Figure 3:**
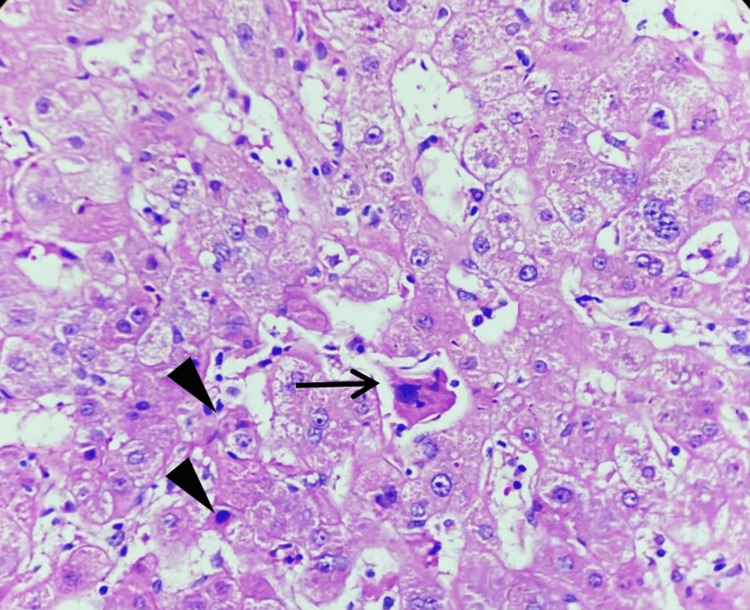
High-power histological section of the liver (H&E). A prominent, immature mononuclear hematopoietic population is observed within the hepatic sinusoids; these cells measure 14-16 µm and feature round, hyperchromatic nuclei with condensed chromatin and scanty basophilic cytoplasm (black arrowheads). Isolated 40-µm megakaryocytes with abundant eosinophilic cytoplasm and irregular, lobulated nuclei are also interspersed throughout the sinusoids (black arrow).

Immunohistochemistry supported the diagnosis of EMH, demonstrating CD61-positive megakaryocytic elements within the sinusoidal and perisinusoidal spaces (Figure 4), along with glycophorin A-positive erythroid precursors. CD31 expression was also documented in megakaryocytic/endothelial elements. No findings compatible with hepatocellular carcinoma, intrahepatic cholangiocarcinoma, or metastatic neoplasm were identified in the analyzed tissue.

**Figure 4:**
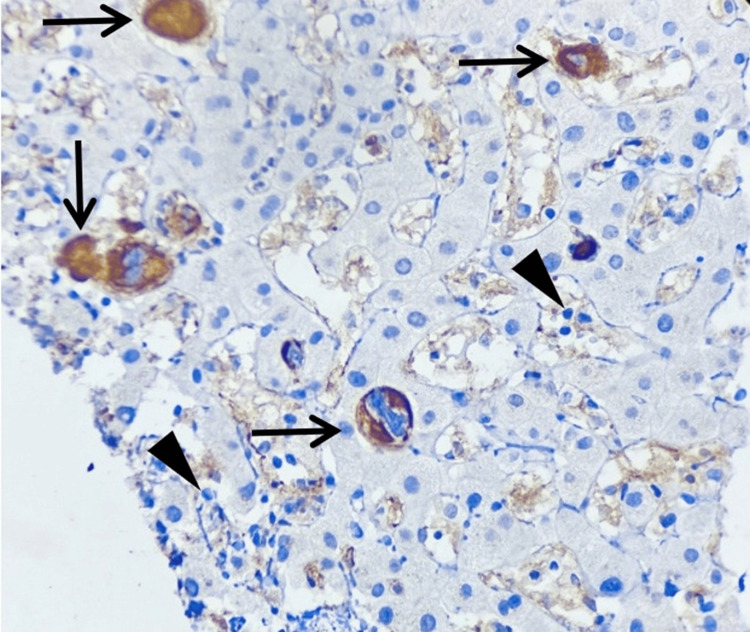
CD61 immunohistochemical staining of liver parenchyma using diaminobenzidine (DAB) chromogen, high-power view. Intense cytoplasmic positivity is observed in large cells located within sinusoidal and perisinusoidal spaces (black arrows), consistent with megakaryocytes. In contrast, the adjacent smaller clusters correspond to erythroid precursors, which are negative for CD61 expression (black arrowheads).

Given the unexpected finding of hepatic EMH, the hematological workup was expanded to include a bone marrow aspirate and biopsy. The bone marrow biopsy showed an approximate cellularity of 40% and grade 1 reticulin fibrosis. These findings, along with the severe anemia, thrombocytopenia, and splenomegaly, were considered suggestive of a possible early-stage myeloproliferative neoplasm, including pre-fibrotic primary myelofibrosis within the differential diagnosis. However, it did not meet sufficient morphological, molecular, or clinical criteria to establish a definitive hematological diagnosis. Further hematologic characterization, including molecular testing for driver mutations commonly associated with myeloproliferative neoplasms, such as JAK2, CALR, and MPL, could not be completed because the patient’s clinical course was subsequently complicated by severe pneumonia, which ultimately led to death.

Based on these findings, primary or metastatic hepatic malignancy was ruled out in the biopsied lesion, and a diagnosis of hepatic EMH manifesting as a space-occupying lesion was established, with radiological and biochemical behavior mimicking a hepatobiliary neoplasm. The case was considered a diagnostic challenge due to its presentation as a suspicious hepatic mass in a patient with chronic liver disease, marked cholestasis, and elevated CA 19-9, in whom a definitive diagnosis of the lesion could only be established through histopathological study.

## Discussion

There are no pathognomonic radiological findings for tumefactive hepatic EMH, and imaging alone is often non-diagnostic [5-6]. Approximately 60% of liver EMH cases are not related to a myeloproliferative neoplasm and occur as a response to pathological stress conditions [5-6]. Newer, actively hematopoietic lesions present a significant diagnostic challenge due to the absence of identifying features such as hemosiderin and fat deposition, causing them to frequently mimic malignant neoplasms on non-invasive imaging. On unenhanced CT, these focal variants typically manifest as well-defined, soft-tissue attenuation masses that can present as either solitary or multiple lesions, commonly coexisting with background hepatosplenomegaly [9-10].

The contrast-enhanced imaging profile of these masses represents a formidable diagnostic challenge across modalities. Both contrast-enhanced CT and gadolinium-enhanced MRI frequently demonstrate a prominent, homogeneous, and persistent enhancement pattern sustained throughout the arterial and portal venous phases [10-11]. Although some lesions may exhibit a heterogeneous enhancement pattern during the initial contrast bolus infusion [4, 7], this predominant hypervascular behavior closely mimics primary hepatic tumors, particularly hepatocellular carcinoma or hepatocellular adenoma [11]. Crucially, a key differentiator on dynamic imaging is that tumefactive EMH characteristically demonstrates persistent enhancement without the classic contrast washout or the delayed capsular enhancement typical of hepatocellular carcinoma, and it lacks delayed enhancement on late-phase imaging [7, 11]. However, exceptional instances of hepatic EMH exhibiting a true contrast washout pattern, thereby perfectly mimicking the classic hallmark of hepatocellular carcinoma, have also been reported [8, 12]. In our case, the diagnostic challenge was further amplified because the lesion did not merely appear as a nonspecific hypervascular hepatic mass; it radiologically simulated hepatocellular carcinoma in a patient with chronic liver disease. Dynamic CT demonstrated heterogeneous arterial-phase enhancement with relatively decreased attenuation on the delayed/parenchymal phase in a 6.3-cm lesion located in segment VIII. In the appropriate clinical context, this enhancement pattern raised strong concern for hepatocellular carcinoma. In addition, the patient had marked cholestasis, a substantially elevated CA 19-9, and a normal alpha-fetoprotein level, which broadened the initial differential diagnosis to include intrahepatic cholangiocarcinoma and other hepatobiliary malignancies. The markedly elevated CA 19-9 level also contributed to the initial suspicion of a hepatobiliary malignancy. However, CA 19-9 is not specific for malignancy and may increase substantially in benign cholestatic conditions, biliary obstruction, cholangitis, and advanced liver disease [13]. Although a direct causal relationship between hepatic EMH and CA 19-9 elevation cannot be established from this case, the finding likely reflected cholestasis and hepatobiliary dysfunction rather than tumor secretion. This reinforces the need to interpret CA 19-9 in the context of cholestasis and to avoid using it as an isolated marker of malignancy. Therefore, histopathological confirmation was essential to avoid misclassification and inappropriate oncologic management. It is also worth noting that despite the significant physical expansion of this hepatic lesion, EMH operates as an incomplete compensatory process that cannot fully supplant or replace definitive marrow function, which explains the persistent severe cytopenias observed in our patient [3].

Another relevant aspect of this case is the direction of the diagnostic sequence. Many reported cases of hepatic EMH occur in patients with a previously established hematologic disorder, especially myelofibrosis or another chronic myeloproliferative neoplasm. In those cases, the appearance of a hepatic mass can be interpreted within an already known hematologic context, and EMH may be considered earlier in the differential diagnosis. In contrast, our patient had no confirmed hematologic neoplasm at the time the liver lesion was detected. The diagnosis of hepatic EMH on liver biopsy was the finding that prompted further hematologic evaluation. Hepatic EMH should not be automatically attributed to myelofibrosis or another clonal hematologic disorder unless diagnostic criteria are fulfilled. At the same time, when hepatic EMH is identified without a known hematologic disease, it should prompt a systematic hematologic workup, including peripheral blood smear, hemolysis profile, iron studies, bone marrow evaluation, and molecular testing when available. In this context, molecular testing has particular diagnostic relevance. Assessment for recurrent driver mutations, including JAK2, CALR, and MPL, should be considered when a myeloproliferative neoplasm is suspected, especially in the presence of cytopenias, splenomegaly, reticulin fibrosis, or unexplained EMH. In our patient, this evaluation would have been necessary to further characterize the suspected early myeloproliferative process; however, it could not be completed because of rapid clinical deterioration and death. Accordingly, in this case, hepatic tumefactive EMH represents the confirmed histopathological diagnosis of the liver lesion, whereas an early myeloproliferative neoplasm remained only a suspected but unconfirmed underlying etiology. Although myeloproliferative neoplasms, chronic hemolytic states, and ineffective hematopoiesis are among the most recognized settings associated with EMH, chronic inflammatory and autoimmune conditions may also influence hematopoietic stem and progenitor cell behavior through persistent immune activation, cytokine-mediated stress, and alterations in the marrow microenvironment [14]. These mechanisms have been described mainly in systemic autoimmune diseases and autoimmune myelofibrosis; however, bone marrow abnormalities have also been reported in association with autoimmune hepatitis [15,16]. In the present case, chronic immune-mediated inflammation related to autoimmune hepatitis may have provided a biologically plausible context for hematopoietic stress, stem cell mobilization, and hepatic EMH. However, because molecular and cytogenetic studies could not be completed, this association remains speculative and should not be interpreted as evidence of a confirmed clonal myeloid disorder.

This reverse diagnostic sequence is one of the most instructive aspects of the present case. The hepatic lesion was initially approached as a possible hepatobiliary malignancy because of the patient’s chronic liver disease, imaging pattern, cholestasis, elevated CA 19-9, and normal alpha-fetoprotein. However, biopsy revealed hepatic EMH, redirecting the diagnostic process toward evaluation for a possible underlying hematologic disorder. This case, therefore, illustrates that tumefactive hepatic EMH may serve not only as a pseudotumor that mimics malignancy but also as a finding that should prompt evaluation for a possible previously unrecognized hematologic process [6].

## Conclusions

Tumefactive hepatic EMH is an uncommon diagnostic entity that may closely mimic primary hepatic malignancy. This case highlights that hepatic EMH may present as a focal liver mass with radiological and biochemical features suggestive of hepatobiliary malignancy, including elevated CA 19-9 and normal alpha-fetoprotein.

In this patient, hepatic EMH was confirmed by histopathological evaluation and immunohistochemistry, with identification of megakaryocytic and erythroid hematopoietic elements and no evidence of hepatocellular carcinoma, intrahepatic cholangiocarcinoma, or metastatic malignancy in the analyzed tissue. Although bone marrow findings raised the possibility of an early myeloproliferative neoplasm, the underlying hematologic disorder remained unconfirmed because definitive molecular and cytogenetic characterization could not be completed. Therefore, hepatic EMH should prompt systematic hematologic evaluation while avoiding premature attribution to a specific clonal disorder without complete diagnostic criteria.
